# Advancements in *ex vivo* machine perfusion for abdominal organ transplantation

**DOI:** 10.3389/frtra.2026.1919998

**Published:** 2026-09-07

**Authors:** Danae G. Olaso, Victoria A. Bendersky, Wyeth D. Alexander, Aleah L. Brubaker, Peter A. Than, Gabriel T. Schnickel

**Affiliations:** Division of Transplantation and Hepatobiliary Surgery, Department of Surgery, University of California San Diego, La Jolla, CA, United States

**Keywords:** hypothermic, machine perfusion (MP), normothermic, organ transplant, perfusion

## Abstract

Organ transplantation remains the definitive treatment for end-stage organ failure, yet its impact continues to be constrained by a persistent shortage of suitable donor organs. Despite advances in surgical technique, immunosuppression, and recipient management, organ preservation has remained relatively unchanged for decades. Machine perfusion (MP) has emerged as a transformative advancement in organ preservation, shifting the paradigm from passive storage to active organ support. By actively circulating perfusate under controlled conditions, MP enables metabolic support, mitigation of ischemic injury, and functional assessment prior to implantation. These modalities collectively represent a paradigm shift from passive preservation toward active resuscitation and repair. This review synthesizes current advances in MP across abdominal organ transplantation, emphasizing the scientific rationale, technical evolution, organ-specific applications, and translational challenges. By examining key clinical trials, emerging biomarkers, and future directions we aim to delineate the roadmap for integrating MP into routine clinical practice and realize its full potential in expanding the donor pool and improving graft outcomes.

## 1 Introduction

Organ transplantation remains the definitive treatment for end-stage organ failure, yet its impact continues to be constrained by a persistent shortage of suitable donor organs. Despite advances in surgical technique, immunosuppression, and recipient management, organ preservation has remained relatively unchanged for decades. Static cold storage (SCS), the historic standard, reduces metabolic demand but does not prevent ongoing cellular injury, provides no opportunity for functional assessment, and offers limited protection against ischemia–reperfusion injury (IRI). These limitations are particularly consequential in the modern era of extended criteria donors (ECD) and donation after circulatory death (DCD), where grafts are more susceptible to ischemic injury and unpredictable post-transplant outcomes.

Machine perfusion (MP) has emerged as a transformative advancement in organ preservation, shifting the paradigm from passive storage to active organ support. By actively circulating perfusate under controlled conditions, MP enables metabolic support, mitigation of ischemic injury, and functional assessment prior to implantation. These modalities collectively represent a paradigm shift from passive preservation toward active resuscitation and repair.

Clinical adoption of MP has expanded rapidly across abdominal organ transplantation, supported by growing evidence demonstrating reduced graft injury, increased organ utilization, and improved logistical flexibility. However, widespread implementation remains limited by challenges in cost, standardization, and the lack of validated biomarkers for organ viability. At the same time, advances in perfusion technology are opening new frontiers, including allograft viability and functional assessment, *ex situ* organ repair, targeted therapeutics, and integration with artificial intelligence.

This review synthesizes current advances in MP across abdominal organ transplantation, emphasizing the scientific rationale, technical evolution, organ-specific applications, and translational challenges. By examining key clinical trials, emerging biomarkers, and future directions we aim to delineate the roadmap for integrating MP into routine clinical practice and realize its full potential in expanding the donor pool and improving graft outcomes.

## 2 Fundamentals of machine perfusion: modalities and timing of intervention

SCS has served as the foundation of organ preservation for over half a century. By reducing temperature, SCS slows cellular metabolism and delays tissue injury; however, it does not halt it. Residual metabolic activity during cold ischemia leads to progressive depletion of adenosine triphosphate (ATP), accumulation of metabolic byproducts, and disruption of cellular homeostasis (1–3). Upon reperfusion, this metabolic debt manifests as ischemia–reperfusion injury (IRI), characterized by oxidative stress, mitochondrial dysfunction, endothelial injury, and activation of inflammatory cascades that contribute to both early and long-term allograft dysfunction (3, 4).

The limitations of SCS impact all potential organs considered for transplant and are amplified in the context of ECD and DCD, where organs are exposed to additional ischemic insult prior to procurement. In this context, SCS provides no reliable mechanism for assessing organ viability prior to transplantation, necessitating clinical decision-making based on incomplete or surrogate data and contributing to high rates of organ discard.

MP was developed to address these fundamental limitations. In contrast with SCS techniques, MP aims to actively circulate perfusate through the organs to maintain microvascular patency, reduce the accumulation of toxic metabolites within the tissue, facilitate endothelial cell integrity and signaling, and provide organ-specific information (vascular flow and resistance) to enable dynamic assessment of organ function prior to transplantation. Conceptually, MP represents a shift from passive preservation to active organ evaluation, resuscitation, and the potential for therapeutic intervention prior to implantation. *Ex vivo* machine perfusion strategies are broadly defined by the temperature and metabolic state at which organs are maintained, reflecting distinct physiologic goals.

### 2.1 Hypothermic machine perfusion (HMP)

Hypothermic machine perfusion (HMP) preserves organs at a low temperature (4 °C and 12 °C) while providing continuous perfusion. Multiple studies have shown that HMP reduces the incidence and severity of IRI and improves post-transplant outcomes (1, 5). HMP preserves mitochondrial activity, maintaining cellular ATP levels, limiting endothelial cell damage, and attenuating reactive oxygen species (ROS) and damage-associated molecular patterns (DAMPs), ultimately reducing oxidative stress and inflammation (2). With these mechanisms combined, non-oxygenated HMP is widely utilized for kidney preservation in the US. Hypothermic oxygenated machine perfusion (HOPE) is the perfusion of an organ with oxygenated preservation solution. In liver allografts, dual hypothermic oxygenated machine perfusion (DHOPE) refers to simultaneous perfusion through both the hepatic artery and portal vein to supply the peribiliary vascular plexes and reduce IRI to the biliary tree. The central tenet of HMP is the continuous perfusion of cold preservation fluid with non-oxygenated and oxygenated systems available for clinical use.

### 2.2 Normothermic machine perfusion (NMP)

Normothermic machine perfusion (NMP) maintains organs at physiologic temperature (34–37 °C), supporting active cellular metabolism. By delivering oxygen, nutrients, and metabolic substrates under near physiologic conditions, NMP enables real-time assessment of organ viability and function (6). NMP enables extended preservation times, permitting transportation across longer distances, accommodation for recipient complexity, and logistical flexibility (7).

### 2.3 Subnormothermic and controlled oxygenated rewarming

Subnormothermic machine perfusion (SNMP) and controlled oxygenated rewarming (COR) are intermediate strategies that transition organs from hypothermic to subnormothermic conditions (20–34 °C) (8, 9). By restoring mitochondrial activity in a controlled manner, they aim to reduce the metabolic stress of abrupt reperfusion; preclinical models of both have demonstrated improved oxygen uptake and increased tissue ATP (9). COR is increasingly used in the clinical “back-to-base” protocol that combines dual hypothermic oxygenated machine perfusion (DHOPE) with NMP, restoring mitochondrial function and minimizing IRI after the period of static cold storage during transport to the recipient hospital (10, 11). Additional studies suggest that SNMP and COR may also reduce inflammation and could help recover marginal grafts or those with prolonged ischemic times (12–15).

## 3 Application of perfusion advances and organ assessment in kidney and liver transplantation

### 3.1 Kidney

The increasing medical complexity of the donor pool has contributed to a rising rate of deceased donor kidney discard, exceeding 28% in the past few years (16). Uncertainty regarding kidney suitability for transplantation and concerns for poor post-transplant outcomes has been a major driving factor for kidney discard. Kidney allograft evaluation has historically relied on donor history, assessment of pre-recovery kidney function, and frozen-section biopsy, which has poor correlation with post-transplant allograft function (17). MP enhances kidney transplantation by improving preservation quality and enabling more informed utilization of marginal grafts. Characteristics of MP, including allograft viability assessment, alleviation of logistical constraints, and metabolic recovery, may help to mitigate kidney discard.

In kidney transplantation, HMP is the most widely adopted MP strategy in clinical practice, with studies demonstrating reduced risk of delayed graft function (DGF) and improved graft survival, particularly for higher risk grafts (16, 17). These benefits have driven formal national adoption across several European countries, especially for ECD and DCD kidneys. The Netherlands demonstrated that national implementation reduced DGF rates from 43.7% to 38.2% (18). Belgium's 2022 national mandate offers perhaps the most compelling evidence: standardizing HMP for all ECD and DCD kidneys not only increased rates of DCD transplantation but generated over 3.5 million euros in healthcare cost savings through reduced dialysis utilization (19). Switzerland has similarly established HMP as the standard of care for DCD and older DBD donors (20). In the United States, national adoption has not occurred, yet approximately 53% of deceased donor kidneys are placed on HMP, with significant variation across transplant centers and OPOs (21). This inconsistency likely leaves clinical and economic benefits unrealized for a meaningful proportion of higher-risk grafts. Oxygenated HMP (HMPO2) represents a promising evolution of this technology, though evidence supporting routine clinical adoption remains limited and ongoing trials will be important in defining its role.

In HMP, parameters such as renal blood flow and vascular resistance are commonly used to guide clinical decision-making. In 2009, a randomized trial of 672 kidneys found that HMP significantly reduced DGF and improved 1-year, 3-year, and 10-year graft survival compared to SCS (18, 19). The LifePort Kidney Transporter used fixed perfusion pressures of 30 mm Hg (18). Additional evidence indicates elevated initial and terminal renal resistance (RR) (≥0.4 mmHg/mL/min) and lower baseline arterial flow (<100 mL/min) predict increased rates of DGF (20–23). Moreover, terminal RR is associated with 5-year graft survival, particularly for moderate and high-KDPI grafts (24). Given this current evidence, no single HMP parameter should drive organ discard; HMP parameters should continue to be combined with clinical variables to guide decision-making.

Centralized assessment centers relocate declined organs away from the transplanting center for ongoing evaluation and have reported comparable rescue rates using distinct perfusion modalities. Emerging data using sub-normothermic acellular machine perfusion (SNAP) is promising. Between 2024 and 2025, 158 deceased donor kidneys declined for transplantation after exhausting standard allocation were evaluated with SNAP at the first centralized kidney Assessment and Repair Center (ARC) in the US (25). Using a non-blood based, acellular, oxygenated perfusate, the kidneys were rewarmed from 8 °C to 35 °C and assessed for transplant suitability over 120 min. Ninety percent were subsequently reallocated and transplanted across 12 U.S. transplant centers with total preservation times extending to 68 h (25). Graft outcomes from this cohort have not yet been reported. In July 2026, Holzner et al. reported 104 declined kidneys rescued following 2 h of NMP at a centralized perfusion service, of which 90% were transplanted and which demonstrated lower rates of delayed graft function than non-NMP kidneys transplanted at the same centers during the study period (26). Centralized assessment therefore appears capable of returning a substantial fraction of declined kidneys to the transplant pool despite markedly extended preservation, though longer follow-up on NMP grafts and any outcome data from ARC-evaluated grafts are needed.

Kidney NMP is emerging as an investigational approach in kidney transplantation, with the potential to provide active metabolic support and real-time functional assessment. NMP reduced renal tubular injury compared to SCS (26). The phase 1 NKP1 trial demonstrated the safety and feasibility of kidney NMP with 100% 30-day graft survival (27). These findings are further supported by data from 13 kidney allografts perfused for a median of 171 min and compared with a matched HMP control group, showing no difference in DGF, serum creatinine, or need for dialysis (28). In DCD donors, NMP was feasible and safe but did not reduce DGF compared with SCS (6). A potential advantage of kidney NMP is the opportunity for functional assessment through urine production and metabolic activity. In the emerging NMP literature, perfusion markers such as arterial flow and vascular resistance, functional markers such as oxygen consumption, lactate, pH, creatinine clearance, ATP, and FMN, and markers of cellular injury such as neutrophil gelatinase-associated lipocalin (NGAL), uromodulin (UMOD), osteopontin (OPN), and tissue inhibitor of metalloproteinases-2 (TIMP-2) may prove important in understanding graft suitability for transplant as well as prognosticating graft longevity. In the kidney NKP1 trial, there were early differences in vascular resistance in kidneys that had functional DGF vs. those that functioned immediately. In addition, markers of renal injury in perfusate were strongly associated with post-transplant outcomes with lower levels of glutathione serum transferase (GST), liver-type fatty acid binding protein (L-FABP), interleukin-18 (IL-18), and lactate dehydrogenase (LDH) in the immediate function group compared to the functional DGF (fDGF) group (27). In pre-clinical porcine models, elevated intrarenal resistance, lower flow, lower oxygen consumption, abnormal acid-base markers, and elevated lactate may predict post-transplant graft function, with a perfusion pressure of 95 mmHg providing the best balance of creatinine clearance and lowest NGAL (29–31). However, applying these markers in combination, and which thresholds reliably predict outcomes, remain unknown; translation of *ex vivo* biomarkers into clinical decision-making in kidney NMP remains limited.

### 3.2 Liver

Liver transplantation has emerged as a leading domain for innovation in MP, driven by the organ's susceptibility to IRI and the need to safely expand utilization of higher-risk grafts. Specifically, liver allografts from DCD donors are associated with increased risk of non-anastomotic biliary strictures due to biliary IRI that leads to narrowing or obstruction of the bile duct (32, 33). MP is increasingly integrated into clinical practice for extended preservation time, organ conditioning and repair, and organ viability assessment to increase organ utilization and reduce organ discard rates particularly for DCD and ECD livers with advanced age, prolonged warm ischemia time (WIT) or high degree of steatosis, whereas traditional static preservation is associated with higher rates of graft dysfunction and biliary complications (3, 34–36). DCD donors rose from 2% in 2000 to 49% of all donors in 2025, driven largely by the widespread adoption of normothermic regional perfusion (NRP) that has increased organ utilization and improved post-transplant outcomes (37–42).

During SCS, organs are injured by direct cold ischemic injury on cell membranes and increased tissue ischemia from hypoxia (43). ECD livers are at increased risk of EAD, PNF, and biliary complications. However, MP mitigates reperfusion injury by reconditioning organs, allowing for organs to be transplanted that would otherwise be discarded. The adoption of MP has led to increased rates of liver graft utilization and in 2023, 12.5% of all livers and over 37% of DCD livers underwent MP preservation prior to transplant (34). MP has led to utilizing livers from older donors or higher body mass index (BMI), with improved post-transplant graft survival (34). The development and adoption of MP has expanded the viability of marginal and ECD liver grafts, such as those from donors of age 60 years or older, prolonged intensive care unit (ICU) admission, elevated liver enzymes, split liver grafts, Hepatitis C, or DCD donors, as well as livers with more than 30%–40% steatosis on biopsy or those with prolonged cold ischemia time (44).

HMP in liver transplantation was first introduced in 2010 when *Guarrera* et al. compared HMP-preserved livers to a matched cohort of SCS-livers and found lower rates of EAD and shorter hospital length of stay with HMP (45). Since its introduction into the field, HMP and HOPE have evolved into a pivotal perfusion advancement. In 2021, a multicenter, randomized controlled trial at six European liver transplant centers compared HOPE with SCS in DCD livers and found that HOPE significantly reduced non-anastomotic biliary strictures, lowered the risk of post-reperfusion syndrome, and decreased EAD (33). This first randomized control trial on HOPE demonstrated significant reduction of ischemic cholangiopathy (IC) through mitochondrial reprogramming under hypothermic aerobic conditions that reduced mitochondrial oxidative injury and IRI-mediated inflammation (33). In 2024, the HOPE-REAL study evaluated long-term outcomes after HOPE in over 1,200 donor livers. The study demonstrated excellent 5-year survival after HOPE-treated DBD and DCD livers with low rates of graft loss secondary to PNF and IC, supporting implementation into clinical practice (46). When paired sequentially in a dynamic perfusion protocol, the DHOPE-COR-NMP protocol saw a lower incidence of non-anastomotic biliary stricture as compared to SCS in a cohort of DCD liver grafts from older donors (11).

In early 2026, HOPE received FDA clearance for commercial use in the United States following the Bridge to Hope trial (NCT05045794). HOPE was associated with reduced EAD, shorter hospital LOS, and less severe biopsy proven rejection compared to SCS liver allografts (47). Early US data include the PILOT trial (NCT03484455) which found that HOPE was safe and noninferior in liver graft preservation compared to SCS (48). The PILOT continued access trial (NCT05574361) found that HOPE was associated with decreased PNF and biliary complications compared with SCS (49, 50).

The current evidence for NMP in liver transplantation is convincing. Since NMP preserves organ function and significantly reduces organ reperfusion injury, NMP has been used to extend preservation times to up to 68 h of *ex vivo* perfusion followed by transplantation without graft rejection or bile injury at 1-year follow up (51). The adoption of NMP has also been shown to reduce organ non-use and discard rates by 50% (7). The Consortium for Organ Preservation in Europe (COPE) was the first multi-center trial across seven European transplant centers to compare NMP to standard SCS in liver transplant in 2016. Of 220 liver transplants, this landmark study demonstrated that NMP was associated with 50% lower levels of graft injury, 50% lower rate of organ discard, and 54% longer average preservation time with no difference in patient or graft survival or biliary complications (7). The 2016–2018 VITTAL trial evaluated 31 discarded livers from UK transplant centers that met high-risk criteria. Livers were perfused on NMP and 71% cleared lactate to ≤2.5 mmol/L within four hours (52). Livers that met viability criteria went on to be transplanted, with 18% developing biliary strictures requiring re-transplantation (52). 5-year patient and graft survival were 82% and 72%, respectively (53). This trial demonstrated that NMP provides the opportunity for expanding organ utilization especially for grafts that exhaust all potential allocation matches.

Two US-based multi-center randomized control trials followed. The PROTECT trial (NCT02522871) compared NMP using the TransMedics Organ Care System (OCS) Liver with SCS across 20 U.S. liver transplant programs (54). Of 300 liver transplants, this trial demonstrated that NMP reduced both post-transplant EAD and ischemic biliary complications and increased organ utilization from DCD donors. There was also no difference in patient and graft survival compared to SCS (54). The second normothermic perfusion trial (NCT02775162) was a multicenter, randomized controlled trial that compared NMP using the OrganOx Metra device to SCS across 15 U.S. liver transplant programs. Of over 260 liver transplants, this study demonstrated no significant difference in rates of EAD between groups and found that NMP had reduced post-reperfusion syndrome and greater effect size in DCD livers from high risk donors compared to SCS, suggesting MP having the greatest benefit for marginal donors (55).

In liver transplant, there is no single best MP modality as each has its individual strengths and direct comparisons between modalities are limited. In a meta-analysis, HOPE/DHOPE was associated with lower risk of EAD, decreased graft failure or loss, and decreased risk of biliary complications compared to SCS while NMP had reduced risk of post-reperfusion syndrome (56). In a meta-analysis of the first seven RCT's, both NMP and HOPE were associated with reduced rates of EAD compared to SCS (36). In the first direct comparison between HMP and NMP, all cause and death-censored graft loss, PNF, and HAT were similar between the MP modalities (57). While HMP improves early allograft function and NMP offers opportunity for viability assessment and organ recovery, additional direct comparisons are needed to elucidate the best MP modality for a liver allograft with varied risks to find the best MP modality for each recipient.

### 3.3 Biomarkers and viability criteria for liver MP

With MP, there have been advances in both perfusate preservation solution and organ viability markers. Preclinical studies evaluating graft-derived cell-free DNA (gdcfDNA), transcriptomic profiling, and microRNA have been studied in the setting of viability assessment during MP and the data may be predictive of organ injury and post-transplant outcomes (58–61). However, molecular profiling remains investigational and needs to be validated before clinical implementation (62). Various studies have proposed differing sets of liver viability assessment on NMP. The VITTAL trial proposed that biomarkers of viability included lactate clearance to <2.5 mmol/L, and two or more of bile production, glucose metabolism, pH > 7.3, hepatic artery flow >150 mL/min portal vein flow >500 mL/min, and homogenous graft perfusion (52, 63). In the DHOPE-COR-NMP trial, bile pH > 7.45, perfusate lactate and pH were biomarkers used to evaluate viability (64). In clinical practice, application of viability assessment remains varied depending on device type and with center-dependent practices and include trending pH, bile production, glucose, lactate clearance, liver enzyme levels and on-NMP histologic assessment. Measuring flavin mononucleotides (FMN), a marker of mitochondrial damage, in the perfusate may also provide real-time data on liver viability (65). In addition to liver NMP, viability criteria have also been investigated in pre-clinical models for kidney NMP (Table 1). Despite these advances, as the field of MP in liver transplantation continues to advance, our evolution of viability criteria will continue alongside a more nuanced understanding of how graft type, device deployment model and recipient factors interplay with emerging biomarkers to develop a composite organ profile.

**Table 1:** Clinical NMP viability criteria.

Organ type	Protocol	Trial	Year	Perfusion approach	Perfusate	Donor type	Device	Criteria
Liver	Birmingham	VITTAL	2016–2019	Back-to-base	Blood-based (pRBC) at 37 °C	DCD + DBD (all UK-declined), *n* = 31	OrganOx metra	Perfusate lactate clearance to ≤2.5 mmol/L within 4 h
								Plus ≥2 of: bile production, glucose metabolism, hepatic arterial flow ≥150 mL/min, portal venous flow ≥500 mL/min, perfusate pH ≥7.30, and/or homogeneous perfusion (52, 63, 66)
Liver	Cambridge		2016–2018	Back-to-base	Blood-based (pRBC) at 37 °C	DCD + DBD (standard + ECD), *n* = 47	OrganOx metra; Liver Assist	Perfusate lactate clearance, ALT levels, supplementary bicarbonate requirements to maintain pH > 7.2, bile pH > 7.5, bile glucose (67, 68)
Liver	Groningen	DHOPE-COR-NMP	2016–2024	Back-to-base with sequential multi-temperature protocol (SCS during transport to recipient center, 1-hr DHOPE at 8–12 °C, 1-hr COR, then NMP at 37 °C)	Hemoglobin based oxygen carrier (HBOC)	DCD only (all nationally declined), *n* = 16	Liver Assist/XVIVO	Perfusate lactate <1.7 mmol/L, pH 7.35–7.45 (64, 69–71)
Kidney	Leicester		2017–2022	Back-to-base	Blood-based (pRBC) at 37 °C	DCD, *n* = 170	Research, modified CPB circuit	EVKP score, macrosopic appearance, flow, urine output, urinary NGAL, KIM-1, endothelin-1, perfusate creatinine clearance (6, 72–74)
Kidney	Groningen		2018–2020	Back-to-base	Blood-based (pRBC) at 37 °C	Porcine + discarded human	Kidney Assist	RBF, UOP, O2 consumption, lactate, pH, AST, LDH, NAG, TIMP-2, H-FABP, FeNA, ATP (75, 76)
Kidney		NKP1	2021–2022	Back-to-base	Blood-based (pRBC) at 37 °C	DCD, *n* = 74	Kidney Assist Transporter	GST-Pi, RBF, UOP, lactate, O2 consumption, perfusate creatinine clearance (27)
Kidney	Innsbruck/Oxford		2022–2024	Back-to-base	Blood-based w/wo urine recirculation	Discarded human	Research/Kidney Assist	NGAL, KIM-1, L-FABP, perfusion parameters, IRR, pH, glucose, lactate, UOP (77–79)

ALT, alanine aminotransferase; MAP, mean arterial pressure; OCS, organ care system; P/F, PaO_2/FiO_2; PVR, pulmonary vascular resistance; EVKP, *ex vivo* kidney perfusion; NGAL, neutrophil gelatinase-associated lipocalin; KIM-1, kidney injury molecule-1; RBF, renal blood flow; UOP, urine output; AST, aspartate aminotransferase; LDH, lactate dehydrogenase; NAG, N-acetyl-*β*-D-glucosaminidase; TIMP-2, tissue inhibitor of metalloproteinases-2; H-FABP, heart-type fatty acid-binding protein; FeNA, fractional excretion of sodium; ATP. adenosine triphosphate; GST-pi, glutathione S-transferase pi; L-FABP, liver-type fatty acid-binding protein; IRR, intrarenal resistance.

### 3.4 Pancreas

MP for pancreas transplant remains largely pre-clinical and experimental. The current standard of care for pancreas transplant is SCS and there are no validated viability criteria for pancreas on MP. In porcine models, pancreas grafts preserved on HMP for up to 6 h had similar recipient survival to grafts on SCS (80). In another study, porcine grafts placed on NMP and transplanted showed graft function and glucose levels within normal range (81). In an early pre-clinical study, six human pancreas grafts perfused on NMP for 4 h were metabolically active and showed minimal injury or edema (82). While MP in pancreas transplant remains largely experimental, there is an ongoing study in the UK using oxygenated HMP for pancreas preservation in the setting of simultaneous kidney-pancreas transplant, and the first pancreas transplanted preserved with oxygenated HMP occurred in mid-2026 (83).

## 4 Emerging directions and innovations

Emerging innovation and pre-clinical models present the potential for exponential growth in MP and organ preservation, reconditioning, and repair. *Ex situ* organ repair using stem cells, gene therapy, or pharmacologic interventions on organs during MP are in the developing stages to improve organ quality for transplantation. The isolated organ on MP is an ideal platform for therapeutic intervention to reduce IRI, improve delivery of genetic therapies, or modulate the immune system to promote tissue repair and recovery while reducing off-target effects (84).

Centralized organ assessment and recovery centers (ARCs) is an emerging platform that offers opportunity for advanced perfusion-based organ viability, preservation, rehabilitation, and therapeutic interventions to expand the donor pool (85). Currently, the UK has established centralized assessment and recovery centers for lung, liver, and kidney allografts declined for transplant with the goal of increasing organ utilization and transplantation (86). In the US, the adoption of ARCs has been limited to lung and more recently, kidney allografts. Centralized lung evaluation systems have expanded access to *ex vivo* lung perfusion (EVLP) that allows for continued evaluation of donor lungs declined for conventional transplantation. Across seven US transplant centers, sixty-six initially declined allografts were ultimately transplanted after EVLP at a centralized lung evaluation system and had similar 30-day and 1-year outcomes compared to conventional lung transplant recipients (87). Continued partnership between OPO's, third party perfusion companies, and transplant centers is needed for the wider adoption of ARCs as a path to evaluate, re-condition, and improve organs declined for conventional transplant.

### 4.1 Mesenchymal stem cells, extracellular vesicles, gene therapy, and pharmacological agents

Mesenchymal stem cells (MSCs), extracellular vesicles (EVs), and gene editing are interesting methods being investigated for therapeutic intervention. MSCs have anti-inflammatory, immunomodulatory, and regenerative properties that aid in organ reconditioning and have been tested in preclinical animal and human kidney models (88, 89). Preclinical studies demonstrate that stem cell-derived EVs reduce organ injury and improve organ function in heart, kidney, liver, lung, and intestinal transplantation models (90). Gene therapy targeting pathways related to inflammation and immunogenicity can reduce the need for systemic immunosuppression and improve long-term graft survival (91, 92).

Pharmacological agents can be added to the standard perfusate during MP to reduce inflammation and IRI or improve organ quality (93, 94). In porcine models, use of anti-inflammatory therapy reduced the inflammatory response of cytokines such as tumor necrosis factor and interleukin-6 (93, 95). Drugs with vasodilatory effect such as prostaglandin E1 or prostacyclin in rodent liver perfusion models showed reduction in markers of hepatic injury (96, 97). Watson et al. observed that occult fibrin and D-dimer release in donor organs was associated with increased rates of liver graft failure and cholangiopathy (98, 99). Fibrinolytic treatment of livers on MP with the addition of alteplase and fresh frozen plasma (FFP) led to increased D-dimer release in perfusate, suggesting the clearance of fibrin, and eight of the nine alteplase treated and transplanted livers were free of cholangiopathy (99, 100). Targeted delivery of antimicrobial drugs on MP to reduce the transmission of infection from donor to recipient can treat infection prior to transplantation. As an example in extra-abdominal organ perfusion, it was possible to treat lung allografts infected with MDRO bacterial and fungal infections that were transplanted without infectious complications (93). As obesity and metabolic dysfunction-associated steatotic liver disease (MASLD) are increasing globally, decreasing steatosis in donor livers is important to improving donor organ quality. Adding defatting agents to the perfusate of livers on NMP have led to significant reduction of hepatocellular lipid content after three hours of perfusion in rat livers, and decreased hepatocyte triglyceride content and macrovesicular steatosis after 6 h of perfusion in human livers (101, 102). In the UK, defatting steatotic liver allografts during NMP using forskolin (NKH477) and L-carnitine reduced intracellular triglycerides (103). These preliminary findings prompted the DeFat Study, an ongoing randomized clinical trial evaluating outcomes in high-risk fatty liver grafts undergoing this fat-removal treatment (104).

Progress toward therapeutic intervention and genetic modification of organs on MP also raises ethical implications of prolonged *ex vivo* perfusion and organ modification. The risks associated with reducing IRI and inflammation, immune modulation, gene therapy, and other drug interventions on organs with prolonged pump times are not well understood (105). The treatment of rejection or post-transplant complications in the setting of these novel therapeutics needs to be considered.

### 4.2 AI and machine learning algorithms in transplantation

Artificial intelligence (AI) and machine learning is being rapidly adopted in cultural and clinical use. A recent study found that AI was able to predict graft failure after liver transplantation (106). Another study used AI to predict progression of DCD donors to reduce futile procurement rates (107). The clinical application and impact of AI in organ transplantation are vast, and AI will likely have a robust application when coupled with MP technology by facilitating advanced organ monitoring, composite allograft risk assessment, and predicting post-transplant outcomes.

## 5 Conclusion

MP has redefined the landscape of organ preservation by introducing dynamic, physiologic, and reparative strategies for graft maintenance. Early success with HMP in kidney transplantation paved the way for the adoption of HOPE/DHOPE and NMP in liver grafts. As the field of MP in abdominal transplantation evolves, areas of focus include refining perfusion approaches and protocols to varying donor and recipient scenarios, improving composite viability assessment, and determining approaches to and timing of therapeutic interventions to improve organ quality. Multidisciplinary collaboration will allow the transplant community to achieve routine, safe, and equitable adoption and advancement of MP in transplantation.
